# Vaxxing to elimination: smallpox vaccines as tools to fight mpox

**DOI:** 10.1172/JCI167632

**Published:** 2023-01-17

**Authors:** Boghuma K. Titanji, Vincent C. Marconi

**Affiliations:** 1 Division of Infectious Diseases, Emory University School of Medicine, Atlanta, Georgia, USA.; 2 Atlanta Veterans Affairs Medical Center, Decatur, Georgia, USA.; 3 Department of Global Health, Emory University, Atlanta, Georgia, USA.; 4 Emory University Vaccine Center, Atlanta, Georgia, USA.

## Introduction

An outbreak of monkeypox (mpox) virus emerged on the global landscape in May 2022 and rapidly spread to several nonendemic countries in every WHO health region. To date, over 80,000 cases have been reported, mostly concentrated in North America, Brazil, and Western Europe (1). After an initial period of relatively rapid spread, which earned the outbreak the designation of a public health emergency of international concern (PHEIC), case numbers have steadily declined, especially in high-income countries. This decline in new cases can be attributed to rapid rollout of vaccines, growing herd immunity built by the rapid spread of infection in individuals considered at highest risk, and behavior modification. There has also likely been a herd effect, with nonvaccinated individuals benefiting from the interruption of transmission among vaccinated individuals.

**Figure 1:**
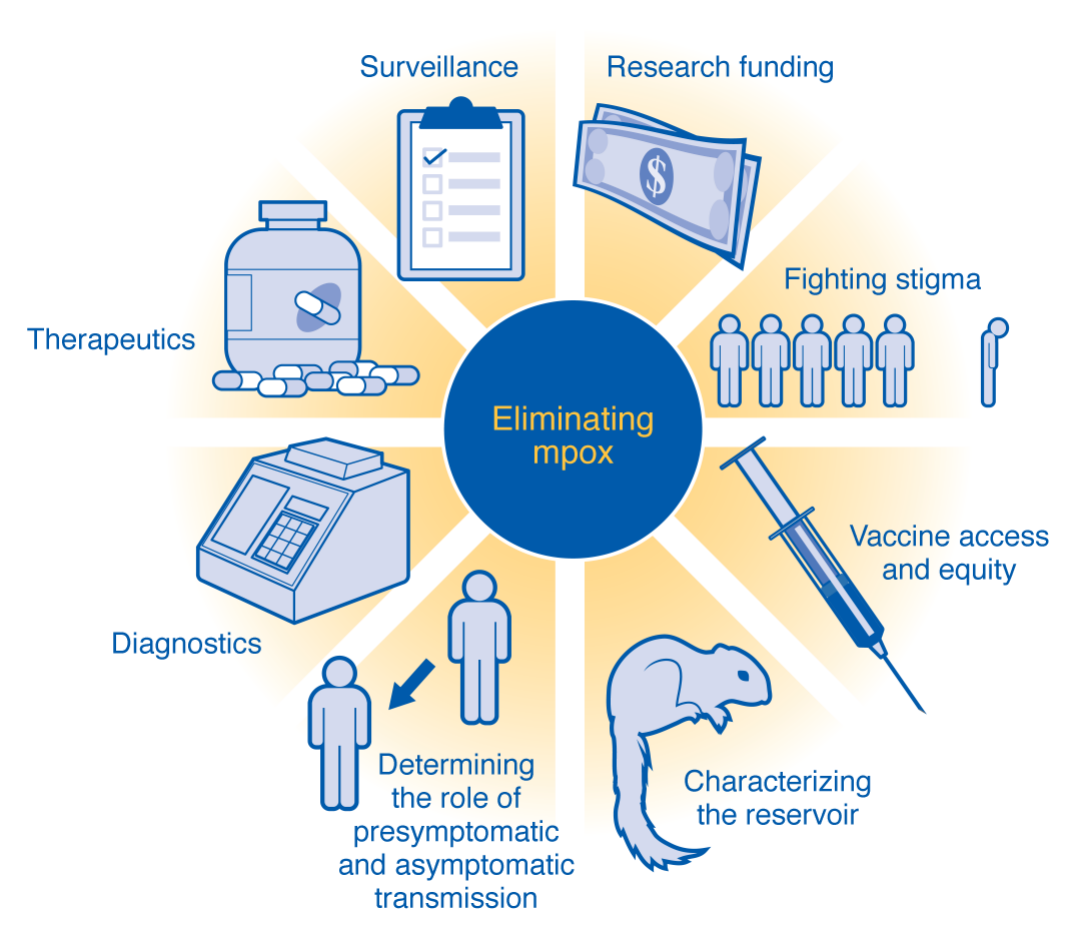
Public health and research considerations for the successful elimination of mpox. Vaccines are a crucial tool for the mpox elimination goal but must be integrated as part of a broader strategy that prioritizes key aspects of research and public health. Surveillance, diagnostics, characterizing the mpox reservoir, understanding the role of presymptomatic and asymptomatic transmission, and research funding for these are important prerequisites for planning. Successful implementation of an elimination strategy must be centered on equitable access to vaccines and therapeutics as well as on fighting stigma and protecting vulnerable communities that are most impacted by mpox.

Much of the response to the mpox outbreak has relied heavily on repurposing smallpox vaccines as tools for pre- and postexposure prevention to contain the outbreak. However, many unanswered questions remain about the role vaccines could play in eliminating mpox as a global threat to human health. Elimination of disease will require a reduction in the number of cases of mpox to zero in defined geographic areas, a goal that may only be feasible in newly affected, nonendemic countries. Eradication is a much more challenging proposition and probably impossible to attain for mpox. While mpox disease is easily diagnosable, the mpox virus has a wide host range, and its reservoir has not been not fully characterized. Moreover, pre- and asymptomatic transmission is a concern, and smallpox vaccines may not provide sterilizing immunity to the disease. In addition, smallpox vaccines are not currently available in mpox-endemic countries in Africa. In this Viewpoint, we discuss what is known about the effectiveness of the available vaccines, emerging data on their use and effectiveness in the current mpox outbreak, and how vaccines fit into the ambitious but more realistic goal of eliminating mpox in newly affected countries and reducing its impact on public health in endemic countries (Figure 1).

## An old foe and with a new phenotype

Mpox virus is a DNA virus belonging to the *Orthopoxvirus* genus and a close relative of variola, which caused the now-eradicated smallpox disease. *Orthopoxviruses* exhibit immunologic cross-reactivity, and immune responses to one *Orthopoxvirus* can confer some degree of protection from infection by other members of the genus (2). During the 2022 global outbreak of mpox, infections have predominantly occurred in men who have sex with men (MSM), representing 90%–95% of cases across cohorts (3, 4). Mucosal transmission through sexual contact has emerged as an important mechanism of transmission and is frequently associated with recto-genital lesions in men, vaginal lesions in women, and pharyngeal lesions in both (3, 5), which had not been described in previous outbreaks. Mucosal immune responses to the mpox virus are not well characterized. Novel routes of virus transmission may have implications for long-term virus shedding or carriage, acquisition of other infections (e.g., HIV), and vaccine responses.

## Preventing mpox with smallpox vaccines

Vaccines made of vaccinia virus, an *Orthopoxvirus* with low virulence, were used to eradicate smallpox and have been shown to confer protection against mpox in several animal models of the disease (6–11). There are currently two licensed smallpox vaccines in the United States. The first-generation vaccines, e.g., Dryvax, were used in the latter phases of the smallpox eradication effort and were phased out for higher-purity, less reactogenic, and better-tolerated second-generation vaccines (12). ACAM2000 (developed by Pasteur Biologics Company) is a second-generation vaccine that consists of a replication-competent, live vaccinia strain derived from a single clonal virus isolate from Dryvax, with reduced neurovirulence in animal models (13). JYNNEOS (developed by Bavarian Nordic) is a third-generation live vaccine containing the modified vaccinia Ankara (MVA) strain, which is replication defective. These vaccines can be used both for preexposure vaccination to prevent infection and as a postexposure strategy for individuals who experience high-risk exposure to mpox. ACAM2000 is administered as a single-dose vaccine using a scarification method to elicit a characteristic “take” at the vaccination site. JYNNEOS is administered as two subcutaneous doses administered 28 days apart. Recently, 20% of the dose given intradermally was authorized by the FDA as a dose-saving strategy on the basis of studies showing equivalent antibody responses compared with standard subcutaneous dosing (14). The immunogenicity of two doses of JYNNEOS is similar to that conferred by a single dose of ACAM2000 and is associated with fewer adverse effects (15). Current CDC guidelines recommend a booster dose two years after the initial series of JYNNEOS and three years after the initial dose of ACAM2000 for individuals with ongoing *Orthopoxvirus* exposure (16). In animal models, live vaccinia vaccines have consistently shown the strongest protection against mpox, with most animals developing sterilizing immunity without symptoms of disease (12). Attenuated vaccines with nonreplicating vaccinia strains also provide strong protection against mpox, but breakthrough symptoms of disease appear to be more common with their use (12). Replication-competent vaccines are contraindicated for immunocompromised individuals (including people with HIV [PWH] with low CD4^+^ T cell counts) and persons with inflammatory skin conditions because of the risk of disseminated vaccinia infections and eczema vaccinatum (12). In the ongoing outbreaks, JYNNEOS has been the preferred vaccine, given its more favorable safety and tolerability profile.

## The unknowns — vaccine effectiveness and durability of protection

Randomized, controlled studies in humans to determine the effectiveness of smallpox vaccines against mpox are lacking. Surveillance studies from the Democratic Republic of Congo (DRC) in the 1980s demonstrated lower attack rates of mpox in individuals previously vaccinated for smallpox when compared with their unvaccinated household contacts, suggesting that the protective efficacy of first-generation smallpox vaccination was approximately 85% (17). In another surveillance study from the mid-2000s, being born prior to cessation of smallpox vaccination in the DRC was associated with a 5.21-fold reduced risk for mpox infection, and the vaccine effectiveness was estimated to be 80.7% (95% CI: 68.2–88.4) (18). There have also been several reports of an attenuated clinical presentation of mpox in previously vaccinated individuals (12). A contemporary vaccine effectiveness study conducted during the current outbreak among active and retired military personnel in the United States, who received smallpox vaccination between 2000 and 2019, estimated the vaccine effectiveness of ACAM2000 at 63% (OR: 0.37; CI: 0.20–0.69), which was similar to Dryvax at 62% (OR: 0.38; 95% CI: 0.17–0.86) (our unpublished observations). These data are reassuring and suggest that a good, although not absolute, level of protection may be conferred by prior smallpox vaccination up to 20 years before exposure. In the current outbreak, a small study in the United States reported that 21 individuals developed symptoms of mpox at least 15 days after receiving one dose of the MVA vaccine, highlighting the importance of two doses to maximize the protective benefit of a third-generation vaccine (19). Another study of healthy participants in the Netherlands showed that in nonprimed individuals, two doses of the MVA vaccine yielded relatively low levels of mpox virus–neutralizing antibodies (20).

The effectiveness and durability of protection conferred by MVA vaccines, which are being used most widely in the ongoing outbreak, need to be evaluated. Moreover, given the prominent role of likely mucosal transmission, mucosal immunity may be important in generating durable immune responses against the mpox virus. It is not currently understood if any of the available vaccines, which are administered through cutaneous routes, confer robust mucosal immunity. Mucosal DCs and IgA secretion at the mucosal interface may be important correlates for mucosal immunity, but their roles need further characterization. Antibodies and cellular immune responses induced by previous smallpox vaccination have been shown to be very durable and remain detectable several decades after vaccination (21, 22). The immune correlates of protection against mpox, however, remain unknown, and waning neutralizing antibody titers over time may be an important consideration for the durability of protection. Indeed, there have been case reports of mpox in individuals with a remote history of smallpox vaccination (23), which may indicate that protection from smallpox vaccines is unlikely to be lifelong against mpox and that boosters may be necessary for individuals with risk factors for ongoing exposure. The timing of such boosters remains to be determined.

## An intersection with HIV and implications for vaccination

Several patient cohorts have shown a strong association between HIV infection and being diagnosed with mpox in the current outbreak. The prevalence of HIV among people with mpox ranges from 30% to 75% across cohorts (3–5, 24). This may reflect behavior patterns in PWH who are healthy, which places them at higher risk of mpox exposure or an increased susceptibility to mpox infection because of their underlying immunodeficiency or both. Additionally, individuals with untreated HIV and advanced immunodeficiency are more likely to have severe manifestations of mpox, require hospitalization for their symptoms, and experience a protracted course of disease (25). Inactivated smallpox vaccines can be safely used in PWH, but their immune responses following vaccination may be less robust than those of healthy individuals. The safety and immunogenicity of JYNNEOS has been evaluated in PWH. In one trial that enrolled individuals with a prior diagnosis of AIDS, who were virologically suppressed and had CD4^+^ T cell counts between 100 and 500 cells/μL, there were no serious safety concerns, and the vaccine was immunogenic at standard dosing (26). In another trial that enrolled PWH with CD4^+^ T cell counts between 200 and 750 cells/μL, vaccine-naive PWH had lower total antibody titers than did the people without HIV at two weeks; however, neutralizing antibody titers were similar in both groups at the 26-week follow-up (27). Furthermore, the total antibody levels were comparable to the titers produced from first- and-second generation live smallpox vaccines in people without HIV. The immunogenicity of inactivated smallpox vaccines in PWH who have CD4^+^ T cell counts below 100 cells/μL or who are not virologically suppressed remains unknown, and whether to repeat the vaccination series or offer additional booster doses once immune reconstitution has been achieved with antiretroviral therapy is a question that needs clarification.

## What about global vaccine equity?

Mpox-endemic countries in Africa have dealt with mpox outbreaks for decades but have mostly been left out of the discourse on vaccination as a strategy to control its spread. Many of these countries do not have access to smallpox vaccines, since widespread vaccination was discontinued following the elimination of the disease. Patterns of vaccine inequity noted during the COVID-19 pandemic have reemerged, and limited doses of smallpox vaccines are currently available only in high-income countries. These inequity hurdles must be overcome to ensure that all who can benefit from these vaccines will have access to them wherever in the world there is a need. Smallpox vaccines were stockpiled by the United States after the eradication of the disease, mainly as part of biowarfare preparedness. Despite the increasing frequency of mpox outbreaks in west and central Africa, the reintroduction of smallpox vaccination as a strategy to combat the disease was never prioritized. In fact, in 2017 the United States destroyed over 20 million doses of expired JYNNEOS vaccine in its national strategic stockpile (28). These doses, in hindsight, could have been better utilized in endemic countries battling mpox.

One can argue that boosting immunity to *Orthopoxviruses* through vaccination of high-risk populations in Africa could potentially have averted the global outbreak by reducing the frequency of outbreaks triggered by zoonotic exposures. Furthermore, poxviruses exist in almost every animal species and present a constant threat for zoonotic spillover events. In reality, prior to 2022, mpox was a severely neglected disease that suffered from a dire lack of funding for disease surveillance, diagnostics, and research to characterize transmission dynamics and zoonotic reservoirs in endemic settings. These are key questions that need to be addressed to inform vaccination strategies in these settings. The spread of mpox in newly affected countries has predominantly occurred through sexual networks of MSM. In outbreaks reported in Africa, transmission has mostly occurred in remote forested areas among household contacts of infected individuals, usually following an initial zoonotic exposure; women and children are frequently affected (2). The varying and unique features of the spread of mpox across global geographies highlights a crucial point; a one-size-fits-all approach to vaccination is unlikely to be effective globally. While it makes sense to offer vaccination broadly to the highest-risk groups (MSM with interconnected sexual and social networks) in newly affected countries, vaccination strategies in endemic African countries may need to be more focused on populations at highest risk for zoonotic exposures. Characterizing emerging sexual transmission among MSM may be especially challenging in settings where homosexuality is criminalized. Success will require an active engagement of the local communities most affected by mpox to help inform policy and also activism to fight the stigma. Besides vaccination, there is also a pressing need for more effective therapies to treat persons with mpox. These efforts will need to be prioritized alongside traditional infection control and prevention strategies to build trust in the communities of those affected by the disease.

## Looking to the future — Is elimination a realistic goal?

It is unlikely that mpox virus, with its wide host range and yet-to-be fully characterized zoonotic reservoir(s), can be eradicated; however, the goal of eliminating mpox as a threat to global public health remains feasible. Success will, however, require sustained investment in research to understand the effectiveness and durability of protection conferred by available vaccines beyond the current outbreak. Importantly, an elimination strategy that is focused only on newly affected, high-income countries is likely to fail. The momentum generated by the emergence of mpox on the global scene must serve as a catalyst to definitively address this once-neglected disease in endemic countries in Africa. Vaccination is a powerful tool that could help accomplish this goal but will need to be combined with improved surveillance, diagnostic tools, and vaccination strategies that are acceptable and adapted to local needs.

## Version 1 01/17/2023

Electronic publication
